# Charges, Reimbursement, and Healthcare Resource Utilization in Patients with Extremity Arterial Injury in the United States: Analysis of Linked Hospital Chargemaster and Claims Data

**DOI:** 10.3390/healthcare14121678

**Published:** 2026-06-12

**Authors:** Elizabeth Brouwer, Fulton Velez, Junwei Tan

**Affiliations:** 1 Curta, Inc., Seattle, WA 98104, USA; 2 Humacyte Global, Inc., Durham, NC 27713, USA

**Keywords:** extremity arterial injury, vascular graft repair, autologous vein graft, hospital charges, healthcare costs, vascular graft infection, limb amputation, healthcare resource utilization (HCRU), claims data analysis

## Abstract

**Highlights:**

**What are the main findings?**

- Infections, amputations, fractures, and post-discharge complications increased costs both before and after discharge.
- Patients treated with autologous vein grafts had fewer outpatient visits, emergency room visits, and hospitalizations than patients treated with other types of grafts.

**What are the implications of the main findings?**

- Informs healthcare decision-makers about the cost of specific complications in patients with extremity arterial injury.
- Helps healthcare decision-makers understand the long-term healthcare resource utilization implications when grafts other than autologous vein are used.

**Abstract:**

Background/Objectives: Successful revascularization following extremity arterial injury is critical for survival and limb salvage. Graft repair is required in ~45% of patients, with the autologous vein preferred for its efficacy and safety. When unavailable, synthetic or non-autologous grafts are associated with infection, amputation, and reduced durability. Extremity arterial injury-specific cost data are lacking, with estimates extrapolated from the general trauma literature. This study characterized the costs and post-discharge healthcare resource utilization (HCRU) for U.S. adults with extremity arterial injury undergoing graft repair. Methods: Adults with extremity arterial injury undergoing graft repair (January 2018 to March 2023) were identified from the linked PINC AI Healthcare Database and Inovalon all-payer claims. Hospitalization charges, costs, and 18-month post-discharge HCRU and costs were assessed. Two-part models estimated cost drivers, adjusted for demographics, clinical characteristics, and complications. Results: Among 964 patients, grafts were autologous (74%), synthetic (14%), other (6%), or multiple (6%). Mean initial hospitalization charges and reimbursed costs were $316,600 and $75,947, respectively. Charges/costs increased with orthopedic fracture (+$639,558/+$91,462), graft infection (+$589,921/+$84,598), and amputation (+$492,986/+$116,611) (all *p* < 0.05). Mean post-discharge costs were $70,222 at 6 months and $93,639 at 18 months. Initial hospitalization complications predicted increased post-discharge costs: orthopedic fracture ($138,683–$145,360) and graft infection ($389,376–$422,224) (both *p* < 0.01). Post-discharge aneurysm, graft infection, and thrombectomy were also associated with higher costs (all *p* < 0.05). Post-discharge HCRU was lowest and most stable with the autologous vein. Conclusions: In-hospital and post-discharge complications are major cost drivers following arterial graft repair. Graft infection was associated with significantly increased costs across both periods, and non-autologous graft use was associated with disproportionately higher 18-month HCRU.

## 1. Introduction

Extremity arterial injury is a serious, life-threatening, and infection-prone type of injury given its high rate of contamination with microbial pathogens [1,2]. Successful revascularization is a critical determinant of both patient survival and limb salvage. In about 45% of cases, patients may require a vascular graft for the repair of a destroyed or poorly viable arterial segment [3]. The gold standard in vascular graft repair is a harvested autologous (native) vein given its short- and long-term performance relative to alternative graft materials, and resistance to infection [4,5,6]. However, the autologous vein is not feasible in about 25% of the cases requiring graft repair due to either unavailability of a suitable vein, poor vein quality, or damage to the vein during trauma [7]. When this is the case, surgeons need to use grafts made of synthetic material or other non-autologous grafts to repair the damaged arterial segment. However, synthetic and other non-autologous types of grafts are associated with a higher risk of complications, such as vascular graft infection, limb amputation, poor patency, and poor durability [8,9,10,11,12,13].

While there is consensus among surgeons and hospital administration staff that vascular complications in graft repair patients prolong the hospital length of stay and generate significant costs, there is currently a dearth of available evidence on the in-hospital and post-discharge costs incurred by extremity arterial injury patients. Specifically, there is a lack of evidence regarding the cost impact of specific complications, and the post-discharge healthcare resource utilization (HCRU) implications of the different graft types used for repair.

There are several studies that estimate the prevalence of vascular injury, common treatment patterns, and related outcomes and complications [5,14], and many publications that take advantage of the Prospective Observational Vascular Injury Trial (PROOVIT) registry, which includes trauma patient treatment and outcome data but not associated costs [15,16]. One publication estimated the costs of initial hospitalization and readmission, comparing open repair in adult trauma patients to endovascular repair, without details of complication-related costs [17]. Further studies are available on the specific costs of limb amputation in trauma patients [18,19,20] and the cost of surgical site infections in general surgery patients [21], but these publications were not specifically tailored to patients with extremity arterial injury.

Proxy cost estimates from other trauma injuries are likely inadequate to inform the costs of care of patients requiring a vascular graft for the repair of a missing or non-viable peripheral arterial segment. Patients with extremity arterial injury usually have a severe clinical presentation with significant blood loss, hypovolemia, varying degrees of ischemia, multi-organ dysfunction, a broad array of concomitant injuries [15,16] (such as venous, neurological and orthopedic injuries), and a propensity for major complications (such as loss of graft patency, aneurysm, tissue necrosis, major amputation, and vascular graft infection) [8,9,10] that can prolong hospital stays and dramatically increase costs.

Updated evidence is needed on the cost of graft repair in adult patients with extremity arterial injury, both during the initial hospitalization and following discharge. Three specific gaps remain: (1) extremity arterial injury-specific cost data have not been reported in a contemporary U.S. population; (2) the incremental cost impact of individual complications such as vascular graft infection, major amputation, rhabdomyolysis, and fasciotomies has not been quantified; and (3) post-discharge HCRU patterns by graft type have not been characterized. These cost estimates, from both a hospital and payer perspective, are essential to understanding the burden of these patients on the healthcare system and to informing resource planning and the adoption of novel technologies in extremity arterial injury. Using real-world data, this study addresses the gaps above by answering three research questions: (1) What are the costs during the initial hospitalization and at 6, 12, and 18 months post-discharge for adult patients with extremity arterial injury treated by graft repair? (2) Which patient-, hospital-, and clinical-level factors drive these costs? (3) How does post-discharge HCRU differ by graft type? Given the descriptive nature of the analysis, the study is framed by research questions rather than formal hypotheses.

## 2. Materials and Methods

This study is a retrospective, cross-sectional analysis of U.S. patients with extremity arterial injury identified in administrative data between 1 January 2018 and 31 March 2023.

### 2.1. Data Source and Patient Population

This analysis was performed using two linked databases: PINC AI^TM^ Healthcare Database (PHD) (Premier Inc. Charlotte, NC, USA), which comprises hospital-based, all-payer information on inpatient discharges, representing approximately 25% of annual United States inpatient admissions; and the Inovalon all-payer insurance claims data (Inovalon, Bowie, MD, USA), which is deidentified and linked with PHD data via secure tokenization. All statistical analyses were carried out using R Version 4.3.3.

Patients were identified from both databases using the following inclusion criteria (Table 1):

- Patient had ≥1 extremity arterial injury occurring in an artery of interest during 1 January 2018–31 March 2023, defined using International Classification of Diseases, Tenth Revision, Clinical Modification (ICD-10-CM) diagnosis codes (arteries of interest include Subclavian, Iliac, Axillary, Brachial, Ulnar, Radial, Hand, Foot, Popliteal, Peroneal, Tibial, and Femoral; Table A1). The first such claim was defined as the patient’s index date. Only the first instance of a diagnosis in a specific artery was identified as a singular injury, with subsequent appearances of codes for arterial injury in the same artery in the following 18 months assumed to be the same injury. Diagnosis codes for injuries in different arteries in the same patient were considered separate injuries.
- Patient had a link key between the datasets to ensure the patient was unique in each database and there was no double-counting.
- Patient age at index date was ≥18 years old.
- Patient injury was treated via definitive open repair using a graft, as defined by Current Procedural Terminology (CPT) and International Classification of Diseases, Tenth Revision, Procedure Coding System (ICD-10-PCS) codes, within 3 days of injury diagnosis code (Table A2). Patients were categorized by the graft type they received based on ICD-10-PCS and CPT codes into the following categories: autologous, synthetic, non-autologous other (e.g., cryopreserved allograft; bovine xenograft), or multiple. Patients categorized as “multiple” must have either received ≥2 types of grafts on the same day for a singular injury (otherwise the graft appearing first in the claims was used for categorization) or had multiple arterial injuries treated with different types of grafts.

### 2.2. Analysis

Baseline demographic and clinical characteristics were summarized in total and by graft type, using basic statistics, including mean and standard deviation for continuous variables and cohort size and percent for categorical variables. Clinical characteristics, such as the Charlson comorbidity index (CCI) and relevant comorbidities, were pulled from up to 6 months prior to the index claim.

**Figure 1:**
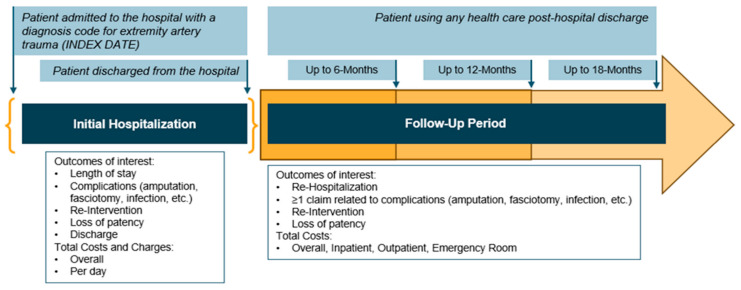
Patient journey schematic.

Cost data were split into two time periods for analysis: (1) the initial hospitalization, and (2) following discharge from the hospital (Figure 1). The initial hospitalization period was defined as admission date to discharge date, encapsulating the qualifying diagnosis code from the inclusion criteria. The post-discharge period was defined as the time span starting the day after discharge from the initial hospitalization and ending 18 months post-injury. The post-discharge period was analyzed at three different time intervals: 6, 12, and 18 months post-injury. Complications occurring during the initial hospitalization or in the follow-up period were defined using CPT and ICD-10-PCS codes (Table A3). Total charges were estimated using PHD hospital data, representing the amount billed for hospital encounters. Total reimbursed costs were estimated using Inovalon claims data, representing the amount the payer paid during the time period of interest. All cost data was inflated to year 2024 based on the medical care consumer price index (CPI) provided by the United States Bureau of Labor Statistics.

HCRU was also estimated in the follow-up periods (6, 12, and 18 months) as the average number of visits (either inpatient, outpatient, or emergency room) per 100 patients. The percentage increase in HCRU from 6 months to 18 months was also assessed.

### 2.3. Statistical Model

Costs and charges were analyzed using a two-part model (logit and generalized linear model with a log link and gamma family) to account for zero-costs and right-skewed data. Initial hospitalization was analyzed in two such models. The dependent variables were (a) total hospital charges and (b) total reimbursed costs from insurance claims. The independent variables in both models were patient age, gender, injury location, graft type, payer type, trauma center level, geographic region, whether there was a concomitant injury (orthopedic fracture, vein injury, nerve injury), and whether a complication occurred (vascular graft infection, limb amputation, rhabdomyolysis, fasciotomy, thrombectomy, and aneurysm).

Post-discharge costs were analyzed in three models with the dependent variable being the total reimbursed costs during each of the 6-, 12-, and 18-month follow-up periods. These models were adjusted for age group, gender, graft type, whether there were concomitant injuries at baseline (orthopedic fracture, vein injury, nerve injury), whether a complication occurred during initial hospitalization (vascular graft infection, limb amputation, rhabdomyolysis, fasciotomy, thrombectomy, and aneurysm), whether a complication occurred during the follow-up period of interest (vascular graft infection, limb amputation, aneurysm, thrombectomy, and endovascular revascularization), and whether the patient had ≥1 inpatient claim, outpatient claim, or emergency room claim during the follow-up period of interest.

## 3. Results

A total of 964 patients were identified for the analysis. The most common graft type used was an autologous graft (74.1%), followed by synthetic (13.8%), non-autologous other (6.0%), and multiple types of grafts (6.1%) (Table A4). The patient population had an average age of 36.5 years (SD 16.2), was 77.2% male, and the majority were covered via Medicaid (50.3%). Those receiving an autologous vein graft were, on average, slightly younger than the other repair groups and had a lower CCI score. Within the total patient population, 53.0% had a concomitant orthopedic fracture, 36.5% had a concomitant vein injury, and 24.1% had concomitant nerve injury encountered during operative repair.

### 3.1. Model Outputs

#### 3.1.1. Average Charges and Reimbursed Amounts

Across 707 patients for whom data were available for initial hospitalization charges, the average amount charged from the hospital for extremity arterial injury with graft repair was $316,600 ± $389,468. Across 465 patients for whom data were available for reimbursed amounts, the average total reimbursement for the initial hospitalization was $75,947 ± $124,729. When standardized by length of stay, the average charge was $29,270 ± $25,996 per day, and the average reimbursed amount was $14,350 ± $36,503 per day (Table A5).

#### 3.1.2. Impact of Demographics on Charges and Reimbursed Amounts

For demographic factors, patients aged 65 and over had significantly higher charges (*p* < 0.01) and significantly lower reimbursement (*p* = 0.02) compared to the reference group (18–49 years old). The trauma center level (other than level I) and geographic region of the hospital (other than the Northeast region) had significantly higher charges accompanied by reimbursed amounts that were lower or only minimally increased (range −$19,648 to $2468; *p* = NS). Across different payer types, there were no significant differences in hospital charges for extremity arterial injury, although average Medicare and Medicaid charges were higher by $271,540 (*p* = 0.48) and $345,845 (*p* = 0.41), respectively, when compared to the reference group (commercial payers) (Table A5). Medicaid and “other” payer types had significantly lower reimbursed cost (−$49,902 and −$39,727, respectively; both *p* < 0.01). Medicare also had lower reimbursement that approached statistical significance (*p* = 0.07) (Table A5).

#### 3.1.3. Hospital Charges by Conduit Type

**Figure 2:**
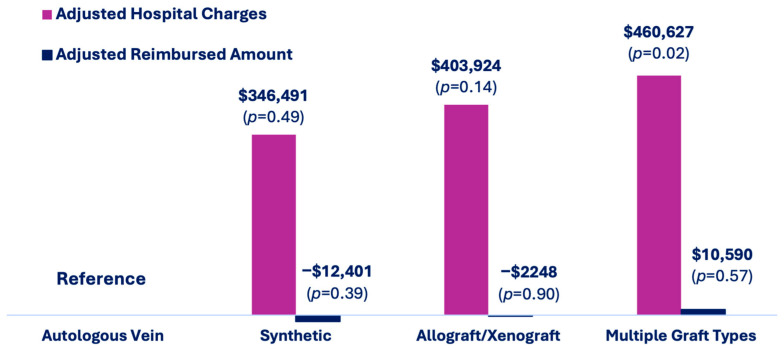
Adjusted average hospital charges and reimbursed amount for the initial hospitalization, by graft type. All costs are inflated to 2024 US dollars.

Adjusted charges were lowest for the autologous vein and higher when other conduits were used. Adjusted charges were $346,491 higher for synthetic (*p* = 0.49), $403,924 higher for non-autologous other (*p* = 0.14), and $460,627 for multiple graft types (*p* = 0.02), compared to the autologous vein reference group (Table A5, Figure 2).

#### 3.1.4. Reimbursed Costs by Conduit Type

Small and non-significant impacts of the conduit type on reimbursement were observed (Table A5). Adjusted reimbursed amounts were lower for synthetic and non-autologous other (−$12,401, and −$2248, respectively), and only marginally higher for the “multiple” category ($10,590), compared to the autologous vein reference group (all *p* = NS) (Table A5, Figure 2).

#### 3.1.5. Hospital Charges by Complication Type

**Figure 3:**
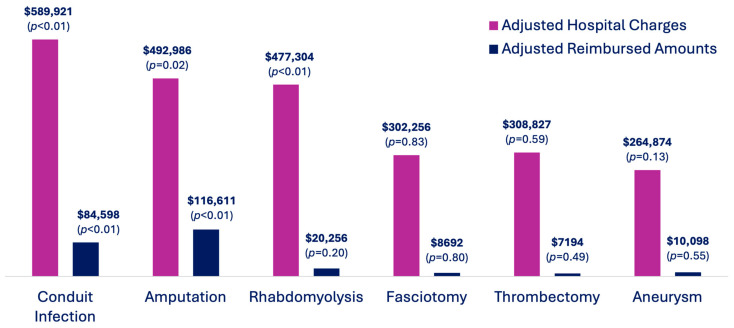
Adjusted change in hospital charges and reimbursed amount for the initial hospitalization, by complication. All costs are inflated to 2024 US dollars.

Complications contributed significantly to hospital charges in extremity arterial injury (Table A5). Vascular graft infection resulted in significant incremental costs of $589,921 during the initial hospitalization, as compared to the no complications reference group (*p* < 0.01), while limb amputation significantly increased charges by $492,986 (*p* = 0.02). Rhabdomyolysis was associated with $477,304 in additional hospital charges (*p* < 0.01), and fasciotomy increased charges by $302,256, though this increase did not achieve statistical significance (Figure 3, Table A5).

#### 3.1.6. Reimbursed Amounts by Complication Type

Vascular conduit infection and limb amputation were associated with significant increases in reimbursed amounts of $84,598 and $116,611, respectively (both *p* < 0.01) (Table A5). Rhabdomyolysis and fasciotomy were also associated with moderate increases in reimbursed amounts ($20,256 and $8692, respectively); however, these did not achieve statistical significance (Table A5).

#### 3.1.7. Impact of Pre-Discharge Clinical Events on Post-Discharge Costs

Post-discharge reimbursed amounts were available for all 964 patients (Table A6). Adjusting for age, sex, and graft type, orthopedic fracture at initial presentation was associated with significantly increased post-discharge costs ($145,360 at 18 months; *p* < 0.01). Vein and nerve injury were also associated with increased costs ($46,575 and $30,818, respectively, at 18 months) but did not achieve statistical significance.

Pre-discharge complications also impacted costs post-discharge. Vascular graft infection during initial hospitalization increased post-discharge costs by $422,224 at 18 months (*p* < 0.01). Pre-discharge rhabdomyolysis was associated with an increase in post-discharge costs of $56,120, but this did not achieve statistical significance. Pre-discharge amputation, thrombectomy, aneurysm and fasciotomy were all associated with lower costs at 18 months, although these differences did not achieve statistical significance.

#### 3.1.8. Impact of Post-Discharge Complications on Post-Discharge Costs

Post-discharge aneurysm, vascular graft infection, and thrombectomy were associated with significantly increased costs at 18 months ($252,331 [*p* = 0.02], $248,430 [*p* < 0.01], and $173,405 [*p* = 0.01], respectively) (Table A6). Post-discharge limb amputation and endovascular revascularization were also associated with increased costs but did not reach statistical significance. Patients with at least one post-discharge inpatient stay claim had increased costs at 6 months that decreased through 18 months, although these did not reach statistical significance, while patients with at least one post-discharge outpatient claim had significantly lower costs at 6 months (−$113,776; *p* = 0.01), 12 months (−$136,733; *p* = 0.01), and 18 months (−$137,860; *p* = 0.02). Patients with at least one post-discharge ER visit claim had increased costs at 6 months ($136,826; *p* = 0.37) and 12 months ($380,654; *p* = 0.09) that further increased through 18 months ($547,459; *p* = 0.04).

#### 3.1.9. Impact of Conduit Type on Post-Discharge HCRU

**Figure 4:**
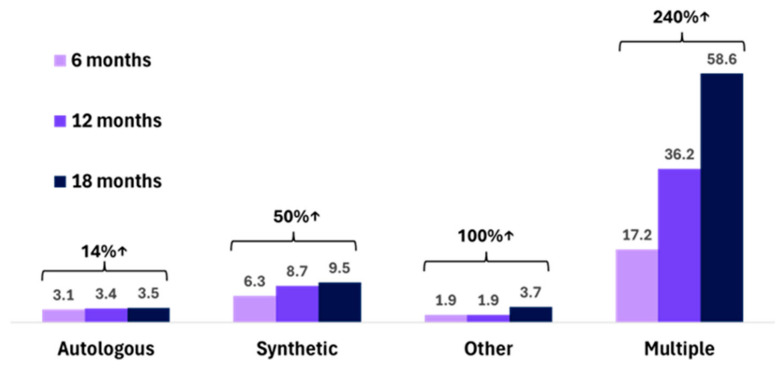
Incidence of post-discharge emergency room visits.

**Figure 5:**
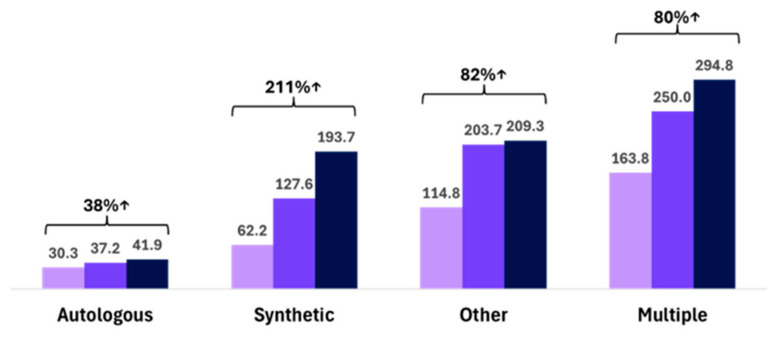
Incidence of post-discharge outpatient visits.

**Figure 6:**
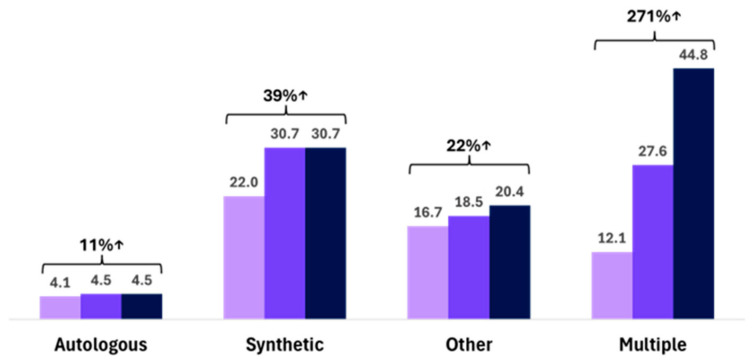
Incidence of post-discharge inpatient stays.

In the overall patient population (i.e., treated with any graft type), patients had an average of 48 outpatient visits per 100 patients in the 6 months post-discharge, as well as 7.8 inpatient visits and 4.3 emergency room visits (Table A7). These increased by 49%, 84%, and 80%, respectively, between 6 and 18 months post-discharge. Compared with other graft types, the absolute rate of HCRU (including inpatient, outpatient, and emergency room use) was lowest with the autologous vein. The increase in HCRU over time was also lowest with the autologous vein (Inpatient: 10.7% increase vs. 22.2–271.4% increase with other conduits; Outpatient: 38.2% increase vs. 80–211.4% increase with other conduits; ER: 14.3% increase vs. 50–240% increase with other conduits) (Figure 4, Figure 5 and Figure 6).

## 4. Discussion

This retrospective claims analysis was conducted to quantify the costs associated with extremity arterial injury that is treated with graft repair, to identify potential cost drivers, and to examine post-discharge HCRU by graft type. The data revealed that treating these patients was expensive, both during the initial hospitalization and in the 6–18 months post discharge, and that HCRU post-discharge was far more stable with the autologous vein than with other vascular graft options.

Average hospital charges for the initial hospitalization approached $320,000, and incremental hospital charges were significantly higher for patients presenting with orthopedic fracture ($640,000), and in patients who developed complications such as vascular graft infections ($590,000) and limb amputations ($493,000) (Table A5). Orthopedic fracture and vascular graft infections also significantly increased post-discharge healthcare costs by $145,360 and $422,224, respectively, at 18 months post discharge (Table A6). Furthermore, the occurrence of late complications (i.e., following discharge from the hospital), such as aneurysm, vascular graft infection, thrombectomy, or any ER visit, also significantly increased costs through 18 months, by $252,000, $248,000, $173,000, and $547,000, respectively (Table A6). Based on these observations, many different complications significantly impact costs. Among the different complications, vascular graft infections are of high relevance from an economic standpoint, in that they consistently generated significant increases in healthcare costs pre- and post-discharge, regardless of their early or late onset.

This analysis also revealed that while there were significant increases in charges with certain conduits and complications, these were not accompanied by proportional increases in reimbursed costs, and in some instances, were accompanied by reductions in reimbursed costs. For example, charges associated with grafts other than the autologous vein increased by more than $340,000, but reimbursed costs decreased for synthetic and non-autologous “other” grafts and increased by just over $10,000 for the “multiple” grafts category (Table A5). Patients > 65 years had significantly increased hospital charges but significantly reduced reimbursed amounts. A trauma center level other than level 1 and geographic region other than Northeast were associated with significantly higher hospital charges (exceeding $340,000), but reimbursed amounts were reduced or experienced only a slight increase. Similarly, patients covered by non-commercial payers incurred greater charges, but had lower reimbursed amounts, which were statistically significant or approached statistical significance (as was the case of Medicare). An injury location other than in the upper extremity, and rhabdomyolysis also significantly increased hospital charges (by more than $400,000 each), but were accompanied by modest, non-significant increases in reimbursed amounts. By contrast, only three variables significantly increased both charges and reimbursed amounts: orthopedic fracture, vascular graft infection, and limb amputation.

One plausible interpretation of this divergent behavior between charges and reimbursed amounts is that hospitals may be under-reimbursed for the additional care these patients require.

This analysis also sheds light on HCRU patterns after discharge from the hospital. Rates of HCRU post-discharge highlight the increased use of healthcare associated with non-autologous graft types, suggesting a link between the type of graft used for repair and post-discharge complexity of care (Table A7).

This analysis had similar findings as a previously published study by Asmar et al., which was a matched cohort study reporting unadjusted cost estimates from the Healthcare Cost and Utilization Project [17]. Our estimated average reimbursed amount per initial extremity arterial injury hospitalization of $75,947 is similar to their observed median cost of open vascular repair of $89,245 in patients with peripheral arterial injury. While that study estimated the unadjusted cost of all readmissions related to the index trauma hospitalization, it did not report on the cost attributable to individual complications. The current analysis reports on the cost impact of specific pre- and post-discharge complications specifically in extremity arterial injury patients requiring repair with a vascular graft.

While autologous vein grafts were associated with lower charges and fewer requirements for follow-up care, a suitable autologous vein may be unavailable for harvest in approximately 25% of patients requiring graft repair. This finding is consistent with prior comparative studies in vascular trauma. Watson et al. [5] reported significantly greater 8-year freedom from graft-related complications with the autologous vein than with expanded polytetrafluoroethylene (ePTFE) in peripheral injuries (77% vs. 31%, *p* = 0.044), and Reilly et al. [11] observed a trend toward higher patency and limb salvage with the autologous vein than with a bovine carotid artery graft (secondary patency 100% vs. 78%; limb salvage 94% vs. 82%). It is well-established in the trauma literature that two types of complications, rhabdomyolysis and need for fasciotomies, are strongly impacted by the speed of revascularization, since more rapid restoration of blood flow to an injured extremity decreases the total ischemia time and lowers the need for fasciotomies and the risk of rhabdomyolysis. The value of new vascular graft options for patients without a feasible autologous vein to harvest may lie in their ability to reduce charges from complications (especially vascular graft infections and limb amputations), shorten the time to revascularization, or both.

Limitations of this study include those inherent in using insurance claims and hospital charge data, which are not produced for the purpose of health services research and therefore may be incomplete or lacking full clinical detail. This limitation is attenuated by using linked datasets that allow for two institutional accounts of each patient’s care. Potential misidentification of patients was also attenuated by combining both ICD-10-CM disease codes with ICD-10-PCS and CPT procedure codes to ensure that patients met inclusion criteria. Low sample sizes for the subgroups of interest (127 synthetic and 54 non-autologous “other” cases), coupled with a wide variability in cost data, likely prevented the identification of a consistent difference in charges and reimbursed amounts for these graft types relative to autologous grafts, despite the very clear separation in post-discharge healthcare resource use trends in this study, and the documented difference in outcomes in the peer-reviewed literature [13]. Lastly, another limitation of this study is the lack of available data regarding ethnicity, co-morbid disease, and trauma severity. The lack of adjustment for these and other potential confounders limits the ability of this analysis to be free of bias.

While a strength of this study is the use of linked chargemaster and healthcare claims data, allowing for a comparison of what is billed vs. what is reimbursed, a limitation is that neither charges nor costs provide a precise measure of the actual cost of patient care. Hospital charges often greatly exceed actual hospital service delivery costs [22], and conversely, reimbursed amounts may be insufficient to cover these true costs. Nevertheless, this study is the first to identify the charges and reimbursed amounts associated with the treatment of extremity arterial injury requiring graft repair. This study also brings into focus opportunities for significant cost savings for trauma centers and payers when complications such as vascular graft infection, limb amputation and rhabdomyolysis can be avoided. Of note, most of the healthcare costs observed in these patients occurred during the initial hospitalization and within 6 months post-discharge, indicating that much of the opportunity for cost avoidance lies within the first several weeks post-injury.

## 5. Conclusions

Vascular graft infections and limb amputations occurring during the initial hospitalization have the greatest impact on the total charges for the initial hospitalization, while vascular graft infections and aneurysms occurring during the initial hospitalization, along with post-discharge vascular graft infections, aneurysms, thrombectomies, and ER visits, had the greatest impact on post-discharge costs. These results highlight the importance of complication prevention and graft selection in extremity arterial injury patients. These findings have important implications for hospitals and payers alike, since investing in cost-effective preventative and/or therapeutic measures could lead to improved patient outcomes while reducing overall healthcare expenditures. The economic case is particularly strong for complication–prevention bundles targeting vascular graft infection given its outsized impact on both initial-hospitalization and post-discharge costs. The post-discharge cost and HCRU advantages of the autologous vein further support continued investment in venous-mapping and conduit-suitability assessment, alongside continued evaluation of graft alternatives that can match the autologous performance when a suitable autologous vein is not available.

## Figures and Tables

**Table 1:** Cohort attrition.

Step	Criteria	N	% of Step Above
Step 1	Unique patients across both PHD and Inovalon databases	819,885	100%
Step 2	Patients in either database with ≥1 diagnosis code (ICD-10-CM) for extremity arterial injury occurring in an artery of interest during 1 January 2018–31 March 2023	22,558	2.8%
Step 3	Patient was ≥18 years old at admission for earliest captured ICD-10-CM code of interest	18,773	83.2%
Step 4	Patient underwent definitive open vascular repair for at least one injury, defined by any relevant procedure code within 3 days of diagnosis AND either (1) no endovascular repair codes or (2) open vascular was the most recent procedure	1618	8.6%
Step 5	Patient received a graft during their definitive open vascular repair procedure	964	59.6%

## Data Availability

The raw data that support the findings of this study are available from the Premier PINC AI^TM^ Healthcare Database and Inovalon all-payer insurance claims data, but restrictions apply to the availability of these data, which were used under license for the current study and are not publicly available. The aggregated data are available from the authors upon reasonable request and with permission of Premier and Inovalon.

## Abbreviations

The following abbreviations are used in this manuscript:

CCI	Charlson comorbidity index
CPI	Consumer price index
CPT	Current procedural terminology
ER	Emergency room
HCRU	Healthcare resource utilization
ICD-10-CM	International Classification of Diseases, Tenth Revision, Clinical Modification
ICD-10-PCS	International Classification of Diseases, Tenth Revision, Procedure Coding System
MD	Maryland
NC	North Carolina
NS	Not significant
PHD	PINC AI^TM^ Healthcare Database
PROOVIT	Prospective Observational Vascular Injury Trial
SD	Standard deviation
U.S.	United States

## Appendix A

Medical codes used for analysis are included in Table A1, Table A2 and Table A3.

**Table A1:** Diagnosis codes by extremity and artery.

Extremity	Artery	Code
UE	Axillary	S45.001A, S45.002A, S45.009A, S45.011A, S45.012A, S45.019A, S45.091A, S45.092A, S45.099A
	Brachial	S45.101A, S45.102A, S45.109A, S45.111A, S45.112A, S45.119A, S45.191A, S45.192A, S45.199A
	Hand	S65.201A, S65.202A, S65.209A, S65.211A, S65.212A, S65.219A, S65.291A, S65.292A, S65.299A, S65.301A, S65.302A, S65.309A, S65.311A, S65.312A, S65.319A, S65.391A, S65.392A, S65.399A
	Other/Unknown	S47.1XXA, S47.2XXA, S47.9XXA, S48.011A, S48.012A, S48.019A, S48.021A, S48.022A, S48.029A, S48.111A, S48.112A, S48.119A, S48.121A, S48.122A, S48.129A, S48.911A, S48.912A, S48.919A, S48.921A, S48.922A, S48.929A, S57.00XA, S57.01XA, S57.02XA, S57.80XA, S57.81XA, S57.82XA, S58.011A, S58.012A, S58.019A, S58.021A, S58.022A, S58.029A, S58.111A, S58.112A, S58.119A, S58.121A, S58.122A, S58.129A, S58.911A, S58.912A, S58.919A, S58.921A, S58.922A, S58.929A, S67.00XA, S67.01XA, S67.02XA, S67.10XA, S67.190A, S67.191A, S67.192A, S67.193A, S67.194A, S67.195A, S67.196A, S67.197A, S67.198A, S67.20XA, S67.21XA, S67.22XA, S67.30XA, S67.31XA, S67.32XA, S67.40XA, S67.41XA, S67.42XA, S67.90XA, S67.91XA, S67.92XA, S68.011A, S68.012A, S68.019A, S68.021A, S68.022A, S68.029A, S68.110A, S68.111A, S68.112A, S68.113A, S68.114A, S68.115A, S68.116A, S68.117A, S68.118A, S68.119A, S68.120A, S68.121A, S68.122A, S68.123A, S68.124A, S68.125A, S68.126A, S68.127A, S68.128A, S68.129A, S68.411A, S68.412A, S68.419A, S68.421A, S68.422A, S68.429A, S68.511A, S68.512A, S68.519A, S68.521A, S68.522A, S68.529A, S68.610A, S68.611A, S68.612A, S68.613A, S68.614A, S68.615A, S68.616A, S68.617A, S68.618A, S68.619A, S68.620A. S68.621A, S68.622A, S68.623A, S68.624A, S68.625A, S68.626A, S68.627A, S68.629A, S68.711A, S68.712A, S68.719A, S68.721A, S68.722A, S68.729A
	Radial	S55.101A, S55.102A, S55.109A, S55.111A, S55.112A, S55.119A, S55.191A, S55.192A, S55.199A, S65.101A, S65.102A, S65.109A, S65.111A, S65.112A, S65.119A, S65.191A, S65.192A, S65.199A
	Ulnar	S55.001A, S55.002A, S55.009A, S55.011A, S55.012A, S55.019A, S55.091A, S55.092A, S55.099A, S65.001A, S65.002A, S65.009A, S65.011A, S65.012A, S65.019A, S65.091A, S65.092A, S65.099A
LE	Femoral	S75.001A, S75.002A, S75.009A, S75.011A, S75.012A, S75.019A, S75.021A, S75.022A, S75.029A, S75.091A, S75.092A, S75.099A
	Foot	S95.001A, S95.002A, S95.009A, S95.011A, S95.012A, S95.019A, S95.091A, S95.092A, S95.099A, S95.101A, S95.102A, S95.109A, S95.111A, S95.112A, S95.119A, S95.191A, S95.192A, S95.199A
	Other/Unknown	S77.00XA, S77.01XA, S77.02XA, S77.10XA, S77.11XA, S77.12XA, S77.20XA, S77.21XA, S77.22XA, S78.011A, S78.012A, S78.019A, S78.021A, S78.022A, S78.029A, S78.111A, S78.112A, S78.119A, S78.121A, S78.122A, S78.129A, S78.911A, S78.912A, S78.919A, S78.921A, S78.922A, S78.929A, S87.00XA, S87.01XA, S87.02XA, S87.80XA, S87.81XA, S87.82XA, S88.011A, S88.012A, S88.019A, S88.021A, S88.022A, S88.029A, S88.111A, S88.112A, S88.119A, S88.121A, S88.122A, S88.129A, S88.911A, S88.912A, S88.919A, S88.921A, S88.922A, S88.929A, S97.00XA, S97.01XA, S97.02XA, S97.101A, S97.102A, S97.109A, S97.111A, S97.112A, S97.119A, S97.121A, S97.122A, S97.129A, S97.80XA, S97.81XA, S97.82XA, S98.011A, S98.012A, S98.019A, S98.021A, S98.022A, S98.029A, S98.111A, S98.112A, S98.119A, S98.121A, S98.122A, S98.129A, S98.131A, S98.132A, S98.139A, S98.141A, S98.142A, S98.149A, S98.211A, S98.212A, S98.219A, S98.221A, S98.222A, S98.229A, S98.311A, S98.312A, S98.319A, S98.321A, S98.322A, S98.329A, S98.911A, S98.912A, S98.919A, S98.921A, S98.922A, S98.929A
	Peroneal	S85.201A, S85.202A, S85.209A, S85.211A, S85.212A, S85.219A, S85.291A, S85.292A, S85.299A
	Popliteal	S85.001A, S85.002A, S85.009A, S85.011A, S85.012A, S85.019A, S85.091A, S85.092A, S85.099A
	Tibial	S85.101A, S85.102A, S85.109A, S85.111A, S85.112A, S85.119A, S85.121A, S85.122A, S85.129A, S85.131A, S85.132A, S85.139A, S85.141A, S85.142A, S85.149A, S85.151A, S85.152A, S85.159A, S85.161A, S85.162A, S85.169A, S85.171A, S85.172A, S85.179A, S85.181A, S85.182A, S85.189A
Other	Iliac	S35.50XA, S35.511A, S35.512A, S35.513A
	Other/Unknown	I97.51, I97.52, I97.618, I97.620, I97.621, I97.638, T81.718A
	Subclavian	S25.101A, S25.102A, S25.109A, S25.111A, S25.112A, S25.119A, S25.121A, S25.122A, S25.129A, S25.191A, S25.192A, S25.199A

**Table A2:** Open vascular graft repair procedure codes by vein type and code type.

Vein Type	Code Type	Code
Autologous	CPT	35521, 35236, 35518, 35522, 35523, 35525, 35566, 35256, 35500, 35556, 35558, 35570, 35571, 35583, 35585, 35587, 35511, 35512, 35516, 35563, 35565, 35682, 35683
	ICD-10-PCS	0317093, 03150A1, 03150A2, 03150A6, 03150A7, 03150A8, 03150A9, 03150AB, 03150AC, 03160A0, 03160A2, 03160A6, 03160A7, 03160A8, 03160A9, 03160AB, 03160AC, 03170A3, 0315090, 0316091, 0317090, 0318091, 0318094, 0319093, 03150A0, 03160A1, 03170A0, 03180A1, 03180A4, 031A094, 031B093, 031C094, 03R507Z, 03R607Z, 03R707Z, 03R807Z, 03R907Z, 03RA07Z, 03RB07Z, 03RC07Z, 03RD07Z, 03RF07Z, 03U507Z, 03U607Z, 03U707Z, 03U807Z, 03U907Z, 03UA07Z, 03UB07Z, 03UC07Z, 03UD07Z, 03UF07Z, 041K09J, 041K09K, 041K0AJ, 041K0AK, 041L09H, 041L09K, 041L0AH, 041L0AK, 041K09H, 041K09L, 041K09M, 041K09N, 041K09Q, 041K0AH, 041K0AL, 041K0AM, 041K0AN, 041K0AQ, 041L09J, 041L09L, 041L09M, 041L09N, 041L09Q, 041L0AJ, 041L0AL, 041L0AM, 041L0AN, 041L0AQ, 041M09L, 041M09M, 041M09Q, 041M0AL, 041M0AM, 041M0AQ, 041N09L, 041N09M, 041N09P, 041N09Q, 041N0AL, 041N0AM, 041N0AQ, 041T09Q, 041T0AQ, 041U09Q, 041U0AQ, 04RK07Z, 04RL07Z, 04RM07Z, 04RN07Z, 04RP07Z, 04RQ07Z, 04RR07Z, 04RS07Z, 04RT07Z, 04RU07Z, 04RV07Z, 04RW07Z, 04UK07Z, 04UL07Z, 04UM07Z, 04UN07Z, 04UP07Z, 04UQ07Z, 04UR07Z, 04US07Z, 04UT07Z, 04UU07Z, 04UV07Z, 04UW07Z, 0313093, 0313094, 0313095, 0313096, 0313097, 0313098, 0313099, 0314094, 031309B, 031309C, 031309J, 031309K, 031309M, 031309N, 03130A2, 03130A3, 03130A4, 03130A5, 03130A6, 03130A7, 03130A8, 03130A9, 03130AB, 03130AC, 03140A0, 03140A2, 03140A6, 03140A7, 03140A8, 03140A9, 03140AB, 03140AC, 041C097, 041C098, 041C09B, 041C09C, 041C09F, 041C09G, 041C09J, 041C09K, 041C0A7, 041C0A8, 041C0AB, 041C0AC, 041C0AF, 041C0AG, 041C0AJ, 041C0AK, 041D096, 041D098, 041D099, 041D09C, 041D09D, 041D09G, 041D09H, 041D0A6, 041D0A8, 041D0A9, 041D0AC, 041D0AD, 041D0AG, 041D0AH, 041D0AK, 041H09B, 041H09C, 041H09F, 041H09G, 041H09J, 041H09K, 041H0AB, 041H0AC, 041H0AF, 041H0AG, 041H0AJ, 041H0AK, 0313090, 0313091, 0313092, 0314091, 03130A0, 03130A1, 03140A1, 03R307Z, 03R407Z, 03U307Z, 03U407Z, 041C096, 041C099, 041C09D, 041C09H, 041C09Q, 041C09R, 041C0A6, 041C0A9, 041C0AD, 041C0AH, 041C0AQ, 041C0AR, 041D097, 041D09B, 041D09F, 041D09J, 041D09K, 041D09Q, 041D09R, 041D0A7, 041D0AB, 041D0AF, 041D0AJ, 041D0AQ, 041D0AR, 041H099, 041H09D, 041H09H, 041H09Q, 041H0AD, 041H0AH, 041H0AQ, 041J09J, 04RC07Z, 04RD07Z, 04RH07Z, 04RJ07Z, 04UD07Z, 04UH07Z, 04UJ07Z
Synthetic	CPT	35621, 35623, 35654, 35266, 35650, 35286, 35656, 35661, 35666, 35671, 35612, 35616, 35663, 35665
	ICD-10-PCS	03150J1, 03150J2, 03150J6, 03150J7, 03150J8, 03150JB, 03150JC, 03160J0, 03160J2, 03160J4, 03160J6, 03160J7, 03160J8, 03160J9, 03160JB, 03160JC, 03150J0, 03160J1, 03170J0, 03170J3, 03180J1, 03180J4, 031B0J3, 03R50JZ, 03R60JZ, 03R70JZ, 03R80JZ, 03R90JZ, 03RA0JZ, 03RB0JZ, 03RC0JZ, 03RD0JZ, 03RF0JZ, 03U50JZ, 03U60JZ, 03U70JZ, 03U80JZ, 03U90JZ, 03UA0JZ, 03UB0JZ, 03UC0JZ, 03UD0JZ, 03UF0JZ, 041K0JK, 041L0JK, 041K0JH, 041K0JL, 041K0JM, 041K0JN, 041K0JQ, 041L0JJ, 041L0JL, 041L0JM, 041L0JN, 041L0JQ, 041M0JL, 041M0JM, 041M0JQ, 041N0JL, 041N0JM, 041N0JQ, 041P0JQ, 041Q0JQ, 041R0JQ, 041S0JQ, 041T0JQ, 041U0JQ, 04RK0JZ, 04RL0JZ, 04RM0JZ, 04RN0JZ, 04RP0JZ, 04RQ0JZ, 04RR0JZ, 04RS0JZ, 04RT0JZ, 04RU0JZ, 04RV0JZ, 04RW0JZ, 04UK0JZ, 04UL0JZ, 04UM0JZ, 04UN0JZ, 04UP0JZ, 04UQ0JZ, 04UR0JZ, 04US0JZ, 04UT0JZ, 04UU0JZ, 04UV0JZ, 04UW0JZ, 03130J1, 03130J2, 03130J6, 03130J7, 03130J8, 03130J9, 03130JB, 03130JC, 03140J0, 03140J2, 03140J6, 03140J7, 03140J8, 03140J9, 03140JB, 03140JC, 041C0J7, 041C0J8, 041C0JB, 041C0JC, 041C0JF, 041C0JG, 041C0JJ, 041C0JK, 041D0J6, 041D0J8, 041D0J9, 041D0JC, 041D0JD, 041D0JG, 041D0JH, 041D0JK, 041H0JB, 041H0JC, 041H0JF, 041H0JG, 041H0JJ, 041H0JK, 03130J0, 03140J1, 03R30JZ, 03R40JZ, 03U30JZ, 03U40JZ, 041C0J6, 041C0J9, 041C0JD, 041C0JH, 041C0JQ, 041C0JR, 041D0J7, 041D0JB, 041D0JF, 041D0JJ, 041D0JQ, 041D0JR, 041F0JB, 041H0J9, 041H0JD, 041H0JH, 041H0JQ, 041J0JJ, 04RC0JZ, 04RD0JZ, 04RH0JZ, 04RJ0JZ, 04UD0JZ, 04UH0JZ, 04UJ0JZ
Other	CPT	35681
	ICD-10-PCS	03150K1, 03150K2, 03150K6, 03150K7, 03150K8, 03150K9, 03150KB, 03150KC, 03160K0, 03160K2, 03160K6, 03160K7, 03160K8, 03160K9, 03160KB, 03160KC, 03170K3, 03150K0, 03160K1, 03170K0, 03180K1, 03180K4, 03R50KZ, 03R60KZ, 03R70KZ, 03R80KZ, 03R90KZ, 03RA0KZ, 03RB0KZ, 03RC0KZ, 03RD0KZ, 03RF0KZ, 03U50KZ, 03U60KZ, 03U70KZ, 03U80KZ, 03U90KZ, 03UA0KZ, 03UB0KZ, 03UC0KZ, 03UD0KZ, 03UF0KZ, 041K0KJ, 041K0KK, 041L0KH, 041L0KK, 041K0KH, 041K0KL, 041K0KM, 041K0KN, 041K0KQ, 041L0KJ, 041L0KL, 041L0KM, 041L0KN, 041L0KQ, 041M0KL, 041M0KM, 041M0KQ, 041N0KL, 041N0KM, 041N0KQ, 041T0KQ, 041U0KQ, 04RK0KZ, 04RL0KZ, 04RM0KZ, 04RN0KZ, 04RP0KZ, 04RQ0KZ, 04RR0KZ, 04RS0KZ, 04RT0KZ, 04RU0KZ, 04RV0KZ, 04RW0KZ, 04UK0KZ, 04UL0KZ, 04UM0KZ, 04UN0KZ, 04UP0KZ, 04UQ0KZ, 04UR0KZ, 04US0KZ, 04UT0KZ, 04UU0KZ, 04UV0KZ, 04UW0KZ, 03130K1, 03130K2, 03130K6, 03130K7, 03130K8, 03130K9, 03130KB, 03130KC, 03140K0, 03140K2, 03140K7, 03140K8, 03140K9, 03140KB, 03140KC, 041C0K7, 041C0K8, 041C0KB, 041C0KC, 041C0KF, 041C0KG, 041C0KJ, 041C0KK, 041D0K6, 041D0K8, 041D0K9, 041D0KC, 041D0KD, 041D0KG, 041D0KH, 041D0KK, 041H0KB, 041H0KC, 041H0KF, 041H0KG, 041H0KJ, 041H0KK, 03130K0, 03140K1, 03R30KZ, 03R40KZ, 03U30KZ, 03U40KZ, 041C0K9, 041C0KD, 041C0KH, 041C0KQ, 041C0KR, 041D0K7, 041D0KB, 041D0KF, 041D0KJ, 041D0KQ, 041D0KR, 041H0KD, 041H0KH, 041H0KQ, 041J0KJ, 04RC0KZ, 04RD0KZ, 04RH0KZ, 04RJ0KZ, 04UC0KZ, 04UD0KZ, 04UH0KZ, 04UJ0KZ

**Table A3:** Codes for outcomes of interest.

Outcome of Interest	Code Type	Code
Comorbidity: Chronic kidney disease	ICD-10-CM	N18.X
Comorbidity: Type 2 diabetes mellitus requiring insulin	ICD-10-CM	E11.X
Concomitant orthopedic fracture	ICD-10-CM	S42.X, S52.X, S02.X, S12.X, S22.X, S32.X, S62.X, S72.X, S82.X, S92.X
Concomitant nerve injury	ICD-10-CM	S04.X, S14.X, S24.X, S34.X, S44.X, S54.X, S64.X, S74.X, S84.X, S94.X
Concomitant vein injury	CPT	34520, 34530
	ICD-10-CM	S25.3X, S25.4X, S25.5X, S25.8X, S25.9X, S35.3X, S35.4X, S35.5X, S35.8X, S35.9X, S45.3X, S45.2X, S45.8X, S45.9X, S55.2X, S55.8X, S55.9X, S65.2X, S65.3X, S65.4X, S65.5X, S65.8X, S65.9X, S75.1X, S75.2X, S75.8X, S75.9X, S85.3X, S85.4X, S85.5X, S85.8X, S85.9X
Limb amputation	CPT	23900, 23920, 23921, 24900, 24920, 24925, 24930, 24931, 24935, 25900, 25905, 25907, 25909, 25915, 25920, 25922, 25924, 25927, 25929, 25931, 26910, 26951, 26952, 27290, 27590, 27591, 27592, 27594, 27596, 27598, 27880, 27881, 27882, 27884, 27886, 27888, 27889, 28800, 28805, 28810, 28820, 28825
Aneurysm	CPT	35011, 35013, 35021, 35022, 35045, 35131, 35132, 35141, 35142, 35151, 35152, 36002
	ICD-10-CM	I72.1X, I72.3X, I72.4X, I72.8X, I72.9X
Fasciotomy	CPT	24495, 25020, 25023, 25024, 25025, 26037, 26040, 26045, 27025, 27027, 27057, 27305, 27496, 27497, 27498, 27499, 27600, 27601, 27602, 27892, 27893, 27894, 28008, 29893
	ICD-10-PCS	0J8.X, 0J9.X
Thrombectomy	CPT	34051, 34101, 34111, 34201, 34203, 35301, 35302, 35303, 35304, 35305, 35306, 35311, 35321, 35351, 35355, 35361, 35363, 35371, 35372, 35875, 35876
	ICD-10-CM	I74.2X, I74.3X, I74.4X, I74.5X, I74.8X, I74.9X, T82.868X
Endovascular revascularization	CPT	35881, 37220, 37221, 37222, 37223, 37224, 37225, 37226, 37227, 37228, 37229, 37230, 37231, 37232, 37233, 37234, 37235, 37236, 37237, 37246, 37247
Vascular graft infection	CPT	35903, 35907
	ICD-10-CM	T81.4X, T82.7X
Rhabdomyolysis	ICD-10-CM	M62.82X, T79.6X

## Appendix B

All analysis results are presented in Table A4, Table A5, Table A6 and Table A7 in Appendix B.

**Table A4:** Baseline demographic and clinical characteristics.

Graft Type	Total		Autologous		Synthetic		Non-Autologous Other		Multiple	
Total Patients	N/Mean	%/SD	N/Mean	%/SD	N/Mean	%/SD	N/Mean	%/SD	N/Mean	%/SD
Baseline Demographic Characteristics										
Age (Mean, SD)	36.5	16.2	34.1	14.2	44.0	19.7	47.8	20.0	38.6	16.2
Age categories (N, %)										
18–49 years	753	78.1%	601	84.2%	77	57.9%	31	53.4%	44	74.6%
50–64 years	141	14.6%	87	12.2%	29	21.8%	14	24.1%	11	18.6%
65 years and over	70	7.3%	26	3.6%	27	20.3%	13	22.4%	4	6.8%
Gender (N, %)										
Female	220	22.8%	148	20.7%	37	27.8%	20	34.5%	15	25.4%
Male	744	77.2%	566	79.3%	96	72.2%	38	65.5%	44	74.6%
Region (N, %)										
Northeast	123	12.8%	93	13.0%	19	14.3%	8	13.8%	3	5.1%
Midwest	273	28.3%	185	25.9%	46	34.6%	22	37.9%	20	33.9%
South	350	36.3%	262	36.7%	41	30.8%	24	41.4%	23	39.0%
West	177	18.4%	146	20.4%	18	13.5%	2	3.4%	11	18.6%
Other	38	3.9%	28	3.9%	7	5.3%	2	3.4%	1	1.7%
Unknown	3	0.3%	0	0.0%	2	1.5%	0	0.0%	1	1.7%
Marital status (N, %)										
Married	123	12.8%	79	11.1%	17	12.8%	17	29.3%	10	16.9%
Single	520	53.9%	405	56.7%	67	50.4%	23	39.7%	25	42.4%
Other	72	7.5%	55	7.7%	12	9.0%	1	1.7%	4	6.8%
Unknown	249	25.8%	175	24.5%	37	27.8%	17	29.3%	20	33.9%
Race categories (N, %)										
White	462	47.9%	338	47.3%	67	50.4%	33	56.9%	24	40.7%
Black	327	33.9%	246	34.5%	45	33.8%	13	22.4%	23	39.0%
Asian	5	0.5%	5	0.7%	0	0.0%	0	0.0%	0	0.0%
Other	88	9.1%	71	9.9%	10	7.5%	4	6.9%	3	5.1%
Unknown	82	8.5%	54	7.6%	11	8.3%	8	13.8%	9	15.3%
Payer categories (N, %)										
Medicare	41	4.3%	17	2.4%	14	10.5%	6	10.3%	4	6.8%
Medicaid	485	50.3%	379	53.1%	64	48.1%	19	32.8%	23	39.0%
Private/commercial	263	27.3%	193	27.0%	32	24.1%	22	37.9%	16	27.1%
Other/unknown	175	18.2%	125	17.5%	23	17.3%	11	19.0%	16	27.1%
Baseline Clinical Characteristics										
Patients with ≥6 months of available claims data prior to index	902	93.6%	668	93.6%	124	93.2%	53	91.4%	57	96.6%
Charlson comorbidity index (Mean, SD)	2.9	2.9	2.3	2.2	4.8	4.1	4.4	3.9	3.4	3.3
Other comorbidities of interest (N, %)										
Chronic kidney disease	26	2.9%	10	1.5%	12	9.7%	2	3.8%	2	3.5%
Type 2 diabetes mellitus	67	7.4%	32	4.8%	20	16.1%	7	13.2%	8	14.0%
Concomitant injuries										
Orthopedic fracture encountered during operative repair (N, %)	511	53.0%	398	55.7%	60	45.1%	24	41.4%	29	49.2%
Vein injury encountered during operative repair (N, %)	352	36.5%	260	36.4%	55	41.4%	20	34.5%	17	28.8%
Nerve injury encountered during operative repair (N, %)	232	24.1%	191	26.8%	24	18.0%	5	8.6%	12	20.3%

SD—standard deviation.

**Table A5:** Initial hospitalization costs and charges.

	Total Charges (N = 707)			Total Costs (N = 465)		
Variables	Mean		SD	Mean		SD
Total cost (raw average)	$316,600		$389,468	$75,947		$124,729
Cost per day (raw average)	$29,270		$25,996	$14,350		$36,503
Model Output	Adjusted Difference	95% CI	*p*-Value	Adjusted Difference	95% CI	*p*-Value
Age						
18–49 (ref group)	-	-	-	-	-	-
50–64	$338,377	(−$1,106,754, $1,772,345)	0.65	−$16,315	(−$39,201, $7110)	0.17
65+	$566,747	($189,466, $925,331)	**0.00**	−$38,000	(−$69,563, −$5183)	**0.02**
Gender						
Female (ref group)	-	-	-	-	-	-
Male	$302,211	(−$501,326, $1,095,777)	0.47	$3878	(−$17,902, $25,531)	0.73
Injury location						
UE (ref group)	-	-	-	-	-	-
LE	$404,567	($116,102, $679,685)	**0.01**	$17,739	(−$7473, $42,365)	0.17
OA	$611,577	($156,017, $1,046,962)	**0.01**	−$16,951	(−$58,771, $25,428)	0.44
Multiple	$477,246	($175,322, $763,425)	**0.00**	$28,713	(−$13,361, $69,841)	0.18
Graft type						
Autologous (ref group)	-	-	-	-	-	-
Synthetic	$346,491	(−$620,469, $1,302,020)	0.49	−$12,401	(−$40,132, $15,738)	0.39
Non-autologous other	$403,924	(−$130,441, $924,963)	0.14	−$2248	(−$37,074, $32,652)	0.90
Multiple	$460,627	($56,995, $849,062)	0.02	$10,590	(−$25,943, $46,774)	0.57
Payer type						
Commercial (ref group)	-	-	-	-	-	-
Medicaid	$345,845	(−$466,024, $1,146,304)	0.41	−$49,902	(−$63,938, −$34,219)	***p* < 0.01**
Medicare	$271,540	(−$469,975, $1,004,096)	0.48	−$35,107	(−$72,452, $3396)	0.07
Other	$365,006	(−$251,175, $969,145)	0.25	−$39,727	(−$60,385, −$17,758)	***p* < 0.01**
Trauma center level						
1 (ref group)	-	-	-	-	-	-
2	$194,223	($119,455, $262,584)	***p* < 0.01**	-$19,648	(−$45,959, $7311)	0.16
3–5	$155,749	($94,335, $212,025)	***p* < 0.01**	-$16,929	(−$54,164, $20,865)	0.38
Region						
Northeast (ref group)	-	-	-	-	-	-
South	$195,701	($109,837, $275,109)	***p* < 0.01**	−$3518	(−$31,830, $24,909)	0.81
West	$222,033	($74,956, $361,785)	***p* < 0.01**	−$5232	(−$37,437, $27,145)	0.75
Midwest	$140,312	($99,459, $176,536)	***p* < 0.01**	$2468	(−$25,321, $30,175)	0.86
Concomitant injuries at baseline—binary, “none” is ref group for each						
Concomitant orthopedic fracture	$639,558	($478,837, $779,179)	***p* < 0.01**	$91,462	($61,732, $118,175)	***p* < 0.01**
Concomitant vein injury	$320,071	(−$7,591,988, $8,221,570)	0.94	$13,514	(−$7556, $34,138)	0.21
Concomitant nerve injury	$331,200	(−$1,562,259, $2,213,733)	0.74	$4948	(−$19,168, $28,901)	0.69
Complications in initial hospitalization—binary, “none” is ref group for each						
Vascular graft infection	$589,921	($247,977, $912,403)	***p* < 0.01**	$84,598	($26,557, $139,848)	***p* < 0.01**
Limb amputation	$492,986	($83,644, $886,063)	**0.02**	$116,611	($50,124, $179,251)	***p* < 0.01**
Rhabdomyolysis	$477,304	($247,361, $691,501)	***p* < 0.01**	$20,256	(−$10,323, $50,166)	0.20
Fasciotomy	$302,256	(−$2,364,483, $2,959,024)	0.83	$8692	(−$56,872, $73,969)	0.80
Thrombectomy	$308,827	(−$794,566, $1,402,031)	0.59	$7194	(−$12,864, $27,015)	0.49
Aneurysm	$264,874	(−$78,047, $599,056)	0.13	$10,098	(−$22,543, $42,405)	0.55

Note: *p*-values derived from two-part generalized linear model, values < 0.05 are in bold. CI—confidence interval; LE—lower extremity; OA—other arteries of interest (subclavian, iliac); Ref—reference group; SD—standard deviation; UE—upper extremity.

**Table A6:** Total healthcare costs post-discharge.

Length of Follow-Up	Total—6 Months			Total—12 Months			Total—18 Months		
Outcome: Total Cost of Follow-Up	Adjusted Difference	95% CI	*p*-Value	Adjusted Difference	95% CI	*p*-Value	Adjusted Difference	95%CI	*p*-Value
Graft type									
Autologous (ref group)	-	-	-	-	-	-	-	-	-
Synthetic	−$38,936	(−$111,142, $34,554)	0.30	−$56,947	(−$134,009, $21,994)	0.16	−$60,891	(−$143,546, $23,773)	0.16
Non-autologous other	$35,191	(−$77,436, $146,657)	0.54	$61,110	(−$73,170, $193,374)	0.38	$51,941	(−$87,928, $190,096)	0.47
Multiple types	−$10,481	(−$108,333, $87,717)	0.84	$24,818	(−$99,028, $147,845)	0.70	$30,157	(−$104,754, $164,073)	0.67
Age group									
18–49 years (ref group)	-	-	-	-	-	-	-	-	-
50–64 years	$41,185	(−$45,616, $126,627)	0.36	$16,479	(−$75,292, $107,707)	0.73	$14,626	(−$82,596, $111,366)	0.77
65+ years	−$12,136	(−$106,200, $82,329)	0.80	−$1705	(−$112,803, $109,450)	0.98	$11,656	(−$113,843, $136,771)	0.86
Gender									
Female (ref group)	-	-	-	-	-	-	-	-	-
Male	−$44,360	(−$95,122, $7865)	0.10	−$61,228	(−$117,010, −$3426)	**0.04**	−$69,051	(−$127,774, −$8050)	**0.03**
Concomitant injuries at baseline—binary, “none” is ref group for each									
Concomitant injuries at baseline: Orthopedic fracture	$138,683	($60,300, $212,490)	***p* < 0.01**	$137,055	($51,922, $217,666)	***p* < 0.01**	$145,360	($53,345, $232,579)	***p* < 0.01**
Concomitant injuries at baseline: Vein injury	$46,929	(−$17,233, $109,542)	0.15	$47,731	(−$23,661, $117,549)	0.19	$46,575	(−$29,819, $121,432)	0.24
Concomitant injuries at baseline: Nerve Injury	−$3286	(−$64,217, $57,753)	0.92	$12,781	(−$59,436, $84,577)	0.73	$30,818	(−$50,290, $110,910)	0.46
Complications in initial hospitalization—binary, “none” is ref group for each									
Complications in initial hospitalization: Vascular graft infection	$389,376	($144,192, $621,714)	***p* < 0.01**	$397,627	($136,395, $645,742)	***p* < 0.01**	$422,224	($137,355, $693,164)	***p* < 0.01**
Complications in initial hospitalization: Limb amputation	$41,633	(−$101,273, $183,166)	0.57	$6634	(−$138,354, $151,403)	0.93	−$3013	(−$154,030, $148,104)	0.97
Complications in initial hospitalization: Thrombectomy	−$16,841	(−$71,701, $38,575)	0.56	−$16,067	(−$79,161, $47,556)	0.62	−$4924	(−$75,360, $65,674)	0.89
Complications in initial hospitalization: Aneurysm	−$83,117	(−$154,236, −$9257)	**0.03**	−$86,473	(−$171,519, $1425)	**0.05**	−$90,060	(−$182,787, $5637)	0.07
Complications in initial hospitalization: Fasciotomy	−$51,251	(−$204,708, $103,896)	0.52	−$82,539	(−$241,365, $79,009)	0.32	−$94,676	(−$261,147, $74,919)	0.28
Complications in initial hospitalization: Rhabdomyolysis	$11,391	(−$65,529, $87,936)	0.77	$53,700	(−$43,282, $148,910)	0.28	$56,120	(−$48,328, $158,716)	0.30
Complications in follow-up—binary, “none” is ref group for each									
Complications in follow-up: Vascular graft infection	$209,040	($98,954, $312,230)	***p* < 0.01**	$248,058	($123,285, $364,647)	***p* < 0.01**	$248,430	($116,483, $372,182)	***p* < 0.01**
Complications in follow-up: Aneurysm	$315,970	($103,708, $517,809)	***p* < 0.01**	$244,511	($45,565, $435,391)	**0.02**	$252,331	($38,251, $458,086)	**0.02**
Complications in follow-up: Limb amputation	$107,164	(−$47,846, $258,639)	0.18	$150,686	(−$23,757, $320,158)	0.09	$102,723	(−$64,305, $266,362)	0.23
Complications in follow-up: Thrombectomy	$195,323	($68,792, $315,411)	***p* < 0.01**	$172,618	($41,421, $298,120)	**0.01**	$173,405	($35,265, $305,825)	**0.01**
Complications in follow-up: Endovascular revascularization	$35,641	(−$129,035, $199,140)	0.68	$71,800	(−$117,040, $258,271)	0.46	$34,899	(−$142,114, $210,761)	0.70
Places of Service—binary, “none” is ref group for each									
Patient had ≥1 inpatient claim	$166,145	(−$131,185, $457,993)	0.28	$84,745	(−$193,312, $360,007)	0.55	$7755	(−$245,723, $260,978)	0.95
Patient had ≥1 outpatient claim	−$113,776	(−$200,846, −$22,953)	**0.01**	−$136,733	(−$233,883, −$35,073)	**0.01**	−$137,860	(−$247,944, −$23,228)	**0.02**
Patient had ≥1 ER claim	$136,826	(−$158,181, $427,318)	0.37	$380,654	(−$63,375, $812,124)	0.09	$547,459	($18,091, $1,058,767)	**0.04**

Note: *p*-values derived from two-part generalized linear model, values < 0.05 are in bold. CI—confidence interval; ER—emergency room; Ref Group—reference group of categorical variables in model.

**Table A7:** Total healthcare resource utilization per 100 patients post-discharge.

	Total		Autologous		Synthetic		Non-Autologous Other		Multiple	
Number of patients	922	100.0%	683	74.1%	127	13.8%	54	5.9%	58	6.3%
	**Mean**	**SD**	**Mean**	**SD**	**Mean**	**SD**	**Mean**	**SD**	**Mean**	**SD**
Follow-up time: 6 months										
Inpatient stays	7.8	71.9	4.1	37.3	22.0	160.8	16.7	77.1	12.1	59.5
Outpatient visits	48.0	377.3	30.3	255.6	62.2	303.1	114.8	578.0	163.8	991.3
ER visits—overall	4.3	41.2	3.1	39.9	6.3	30.2	1.9	13.6	17.2	77.5
Follow-up time: 12 months										
Inpatient stays	10.4	104.4	4.5	39.7	30.7	246.1	18.5	89.2	27.6	119.6
Outpatient visits	72.8	556.6	37.2	309.0	127.6	606.0	203.7	1035.0	250.0	1409.1
ER visits—overall	6.1	59.2	3.4	41.6	8.7	50.4	1.9	13.6	36.2	170.3
Follow-up time: 18 months										
Inpatient stays	11.6	112.7	4.5	39.7	30.7	246.1	20.4	101.6	44.8	201.0
Outpatient visits	88.5	673.2	41.9	345.3	193.7	1030.5	209.3	1043.8	294.8	1558.3
ER visits—overall	7.8	86.6	3.5	41.8	9.4	52.6	3.7	19.1	58.6	301.5
Increase in incidence rates from 6 to 18 months										
Inpatient stays	48.6%		10.7%		39.3%		22.2%		271.4%	
Outpatient visits	84.2%		38.2%		211.4%		82.3%		80.0%	
ER visits—overall	80.0%		14.3%		50.0%		100.0%		240.0%	

ER—emergency room; SD—standard deviation.
